# Phenotypic and genomic characterisation of performance of tropically adapted chickens raised in smallholder farm conditions in Ethiopia

**DOI:** 10.3389/fgene.2024.1383609

**Published:** 2024-04-19

**Authors:** Katrina M. Morris, Kate Sutton, Mekonnen Girma, Enrique Sánchez-Molano, Bersabhe Solomon, Wondmeneh Esatu, Tadelle Dessie, Lonneke Vervelde, Androniki Psifidi, Olivier Hanotte, Georgios Banos

**Affiliations:** ^1^ The Roslin Institute, University of Edinburgh, Midlothian, United Kingdom; ^2^ International Livestock Research Institute (ILRI), Addis Ababa, Ethiopia; ^3^ Royal Veterinary College, Hatfield, United Kingdom; ^4^ School of Life Sciences, University of Nottingham, Nottingham, United Kingdom; ^5^ Scotland’s Rural College (SRUC), Animal and Veterinary Sciences, Midlothian, United Kingdom

**Keywords:** chicken, sub-Saharan Africa, phenotyping, genomics, smallholder, village

## Abstract

**Background:**

In sub-Saharan Africa, 80% of poultry production is on smallholder village farms, where chickens are typically reared outdoors in free-ranging conditions. There is limited knowledge on chickens’ phenotypic characteristics and genetics under these conditions.

**Objective:**

The present is a large-scale study set out to phenotypically characterise the performance of tropically adapted commercial chickens in typical smallholder farm conditions, and to examine the genetic profile of chicken phenotypes associated with growth, meat production, immunity, and survival.

**Methods:**

A total of 2,573 T451A dual-purpose Sasso chickens kept outdoors in emulated free-ranging conditions at the poultry facility of the International Livestock Research Institute in Addis Ababa, Ethiopia, were included in the study. The chickens were raised in five equally sized batches and were individually monitored and phenotyped from the age of 56 days for 8 weeks. Individual chicken data collected included weekly body weight, growth rate, body and breast meat weight at slaughter, Newcastle Disease Virus (NDV) titres and intestinal Immunoglobulin A (IgA) levels recorded at the beginning and the end of the period of study, and survival rate during the same period. Genotyping by sequencing was performed on all chickens using a low-coverage and imputation approach. Chicken phenotypes and genotypes were combined in genomic association analyses.

**Results:**

We discovered that the chickens were phenotypically diverse, with extensive variance levels observed in all traits. Batch number and sex of the chicken significantly affected the studied phenotypes. Following quality assurance, genotypes consisted of 2.9 million Single Nucleotide Polymorphism markers that were used in the genomic analyses. Results revealed a largely polygenic mode of genetic control of all phenotypic traits. Nevertheless, 15 distinct markers were identified that were significantly associated with growth, carcass traits, NDV titres, IgA levels, and chicken survival. These markers were located in regions harbouring relevant annotated genes.

**Conclusion:**

Results suggest that performance of chickens raised under smallholder farm conditions is amenable to genetic improvement and may inform selective breeding programmes for enhanced chicken productivity in sub-Saharan Africa.

## 1 Introduction

Accelerated food demands fuelled by a fast-growing human population and increased average purchasing power are putting pressure on the food supply chain worldwide. The situation is especially challenging in low- and medium-income countries (LMIC), where food security features as an issue of paramount importance (Barrett, 2010). The Food and Agriculture Organisation of the United Nations estimates that food production must at least double in LMIC by the year 2050 (FAO, 2014) to meet demand and meaningfully contribute to hunger alleviation.

Thanks to their adaptability, versatility and ease of handling, chickens have become a key source of high-quality protein worldwide. The global number of chickens more than doubled in the period 1990–2021 and poultry is now the most produced type of meat in the world accounting for nearly 40% of all meat production (Statista, 2023).

The increasing importance of poultry in LMIC in sub-Saharan Africa is well documented (Malatji et al., 2016; Statista, 2022). Chickens account for more than 98% of all poultry species in the continent (Hassen et al., 2006) and numbers, production levels and demand increase fast. About 80% of chicken production in Africa emanates from birds kept outdoors in smallholder village farms, where they range freely in low input, (semi) scavenging conditions (Sonaiya, 2008; Khobondo et al., 2015).

Despite advances in the management and selection of commercial chickens in intense production environments (Zuidhof et al., 2014; Wolc et al., 2016), genetic improvement of chickens adapted to smallholder farming in LMIC has not achieved the same level of attention and progress. The necessity for locally tailored breeding programmes has already been documented (Bettridge et al., 2018), but the lack of accurate data on individual chicken performance on key traits of interest to smallholder farmers constitutes a major setback. International initiatives, including the Tropical Poultry Genetic Solutions (TPGS; Dessie, 2021) and its predecessor, the African Chicken Genetic Gains (ACGG, 2019) programmes, have been put in place to this effect, providing services and tools for increased smallholder chicken productivity and sustainability. In this context, the need for careful and accurate phenotypic and genetic characterisation of tropically adapted chickens has been recognised (Dessie et al., 2011). Understanding the phenotypic dynamics and genomic background of performance of chickens reared in smallholder farm conditions are necessary steps for long-term sustainability and improvement.

The present study set out to (i) perform a large-scale phenotypic characterisation of tropically adapted commercial chickens reared in smallholder farm conditions for performance traits linked to growth, meat production, immunity, and survival, and (ii) examine the genetic profile of these traits. We focused our study on the Sasso chicken, a dual-purpose commercial type that is becoming popular amongst smallholder poultry farmers in sub-Saharan Africa (Aman et al., 2017). Previous studies have addressed the suitability of Sasso for a wide range of tropical systems (Yakubu and Ari, 2018) and agroecological zones (Aman et al., 2017). Combinations of different parental lines originating mainly in France generate multiple Sasso crosses today. The specific chicken type of the present study, Sasso T451A, is a cross between T44 males (https://africa.sasso-poultry.com/en/sasso-products/colored-chickens/male-breeders/ruby-t/; Tola et al., 2022) and S51A females (https://africa.sasso-poultry.com/en/sasso-products/colored-chickens/female-breeders/sa51a/; Duy Hoan et al., 2023). The Sasso cross T451A has been introduced to sub-Saharan Africa as a slow-growing chicken suitable for raising in smallholder village conditions (Getiso et al., 2017; Yakubu and Ari, 2018; ACGG, 2019). Importantly, the prompt availability of a sufficient number of 1-day old Sasso T451A chicks underpinned our objective for a large-scale research study.

## 2 Materials and methods

### 2.1 Animals and sampling

The present study included a total of 2,573 Sasso T451A chickens reared at the poultry facility of the International Livestock Research Institute (ILRI) in Addis Ababa, Ethiopia. The site’s geographic coordinates are 9^o^1′48 N and 38^o^44′ E on an elevation of 2,382 m.

To allow proper monitoring, observation, and detailed recording of individual birds, chickens were raised in five batches of approximately equal size (507–520 birds), between 21 October 2019 and 18 February 2021.

In the first phase of each batch, birds were procured as 1-day old chicks from EthioChicken, a private poultry breeding company. The chicks were individually wing-tagged and reared indoors in deep litter brooding conditions until the age of 56 days. During this phase, all chickens were vaccinated according to the recommendations of the National Veterinary Institute of Ethiopia (NVI, 2016; Supplementary Table S1). For logistic reasons associated with the COVID-19 pandemic, the indoor brooding phase took place in EthioChicken farms for the first two batches and at the ILRI poultry facility for the remaining batches. The same procedures for bird management, vaccination, feeding, and handling were applied in all cases.

The second phase constituted the main phase of the present study and started when, at 56 days of age, birds were moved outdoors to specially designed paddocks at the ILRI poultry facility, emulating free-ranging conditions in smallholder farms. Average stocking density was 0.7 birds per square metre, within the recommendation scale for free-ranging chickens (https://www.freedomrangerhatchery.com/blog/square-feet-per-chicken/). The age of 56 days was chosen to represent the typical age that birds are sold to village smallholders by the breeding companies. During this outdoor phase, chickens ranged freely and were fed following the Sasso recommendations for growing chickens aiming to encourage scavenging behaviour (https://africa.sasso-poultry.com/en/; SASSO FGS, 2018). Feed supplementation was according to chicken body weight at each stage of growth and included a daily average of 55–85 g of feed per bird with a protein content of 18% and metabolizable energy of 2,900 kcal/kg of feed. The outdoor phase lasted 8 weeks to allow the chickens to grow towards an average typical target market body weight of approximately 1.5 kg. Exceptionally, the first batch only lasted 4 weeks for operational reasons. Data collection on individual birds for the purposes of the present study took place during this second phase outdoors, which hereafter will be referred to as the monitoring period.

On the first day of the monitoring period, blood from the wing vein and cloacal swab samples were collected from all chickens. One hundred μl of blood was conserved in ethanol and the remaining blood was left to coagulate overnight for serum collection.

Body weight of each chicken was measured and recorded on the first day of the monitoring period and weekly thereafter. On the last day of the monitoring period chickens were weighed and slaughtered, and the breast muscle was excised and weighed. At the same time, blood and cloacal samples were collected again.

The procedures for chicken handling, monitoring, sampling and recording had already been developed and streamlined before the first batch, in a pretrial batch of approximately 120 different chickens of the same type. Data collected during the pretrial batch informed decision-making and good practice for the main study but were not considered in the ensuing data analyses.

### 2.2 Animal phenotyping

#### 2.2.1 Growth and meat production traits

For each chicken, the growth rate was calculated as grams gained per day based on weekly body weight records during the monitoring period. Three growth rate traits were considered: growth in the entire monitoring period; early growth during the first 4 weeks of the monitoring period; and late growth during the last 4 weeks of the monitoring period.

Three meat production traits were considered for each bird: body weight at slaughter, breast muscle weight, and proportion of breast muscle to body weight.

#### 2.2.2 Immune and survival traits

Blood samples collected from the pretrial chickens at the end of the test batch were initially used to examine antibody titres specific for Infectious Bronchitis Virus (IBV), Newcastle Disease Virus (NDV) and Avian Influenza Virus (AIV). Substantial variation in NDV titres was observed among these chickens, implying that high and low immune responders would be possible to identify in the field. On the contrary, no variation was observed in titres for IBV and AIV.

Based on these findings, blood samples collected on each chicken of the main study on the first and last day of the monitoring period were used to derive antibody titres for NDV. Blood samples were first allowed to coagulate at room temperature before removal of the serum. Serum samples were subsequently stored at −20°C. Serum NDV-specific titres were analysed using commercial IDEXX NDV ELISA kits (BioChek BV, Reeuwijk, Netherlands; serum dilution 1:100), according to manufacturer’s instructions, as previously described (Girma et al., 2023). Three phenotypes were then recorded for each bird: NDV titre at the beginning of the monitoring period; NDV titre at the end of the monitoring period; and difference between NDV titres at the beginning and end of the monitoring period. The first NDV measurements mainly reflected chicken variation in vaccine response during the indoor brooding phase, whereas the other two measurements also reflected response to natural exposure to endemic NDV during the outdoor monitoring period in the field.

Cloacal samples collected on the first and last day of the monitoring period were used to measure mucosal total Immunoglobulin A (IgA) levels on each bird. These samples were retrieved using FloqSwaps (Copan no. 552C, California, United States) and were subsequently placed in 500 μL of PBS and stored at −20°C. Total IgA measurements were derived from these samples using a direct in-house developed sandwich ELISA. In brief, 96-Well Nunc MaxiSorp^®^ flat-bottom plates were coated overnight at 4°C with mouse anti-chicken IgA (Clone A-1, IgG2b, 1 μg/mL, Southern Biotech, United States) diluted in coating buffer (50 mM bicarbonate in H20; Sigma-Aldrich, Gillingham, United Kingdom). The plates were blocked (10% casein; Sigma-Aldrich, Gillingham, United Kingdom) at RT for 1 h. Cloacal samples were diluted at 1:50 in blocking buffer and added to the plates in duplicate for 1 h at RT. Plates were washed 3 times (PBS and 0.5% Tween-20; Sigma-Aldrich, Gillingham, United Kingdom) before adding the detection antibody, goat polyclonal anti-chicken IgA-HRP (IgG, 0.25 μg/mL; Biorad, California, United States). Subsequently, one-step ultra TMB (ThermoFischer Scientific, Paisley, United Kingdom) was added to each well and reaction was allowed for 10 min before quenching with 2 M sulfuric acid (Sigma-Aldrich, Gillingham, United Kingdom). For each step, 50 µL were added to each well and 200 µL were used for all washing steps. The absorbance was measured at 450 nm using a microplate reader. Each plate consisted of a negative control (no primary antibody), a positive control from the supernatant of homogenised intestinal tissue from a 4-week-old Hy-line chicken, and a no-sample control. All ELISA data were normalised to the no-sample control present on each plate.

As with NDV, three IgA phenotypes were recorded for each chicken: IgA levels at the beginning and end of the monitoring period, and the difference between the two measurements. These IgA phenotypes collectively reflected variation between birds in mucosal response to infection.

Finally, a survival phenotype was recorded for each chicken as a binary trait denoting whether the bird survived to the end of or perished during the outdoor monitoring period. Reason of death was also recorded following post-mortem examination, when known.

### 2.3 Animal genotyping by sequencing

One hundred μl of the blood drawn on the first day of the monitoring period was preserved in cryo-tubes filled with 1.5 mL absolute ethanol (100%). Samples were transferred into QIAcard FTA Elute Micro cards (Sigma-Aldrich, Gillingham, United Kingdom) for transport to the sequencing provider facility, Neogen Genomics (Nebraska, United States), for low-pass whole-genome sequencing at 0.5X coverage. Imputation of Single Nucleotide Polymorphism (SNP) markers for all samples was then performed by Gencove (New York, United States) with proprietary software. Imputation was performed using an existing panel of 583 high-coverage (30X) chicken genomes comprising multiple samples including the Sasso T451A bird of the present study, mapped to the *Gallus gallus* six genome. The imputation panel density was approximately 28.9 million variants.

The accuracy of the imputation process was assessed using two methods. Firstly, a down-sampling approach was followed, where individual samples from the imputation panel were sequentially removed from the panel, their low-pass genotype was then included in the imputation to the reduced panel, and the correlation between imputed and actual high-coverage sequences was estimated. The process was repeated for 12 imputation panel samples. Secondly, whole-genome sequencing in high coverage (20X) was performed on 20 of the samples from the present study (four randomly selected samples from each batch). Quality of fastq files from the high-coverage sequencing was checked with FastqQC (Andrews, 2010) and SNPs were called using the GATK best practices pipeline (Van der Auwera and O’Connor, 2020). Concordance between imputed datasets and SNPs called on high-coverage whole-genome sequences was performed with VCF-comp (https://github.com/tmoerman/vcf-comp).

Of our 2,573 chicken samples, imputed sequencing data were derived for 2,476. For the remaining 97 (3.77% of the total) the DNA sample quality was too poor for sequencing or imputation. The following quality control edits were subsequently applied. Indels, non-biallelic SNPs and SNPs with minor allele frequency lower than 0.02 were removed. SNPs with low genotype probability corresponding to a genotyping score lower than 0.90 were removed and recorded as missing data. Similarly, samples missing more than 10% of the genotypes were removed. The remaining SNPs were then pruned for linkage disequilibrium using pair-wise genotype correction (*r* ^2^ > 0.80) in 100-SNP sliding windows with a step of 10 SNPs across the genome. Identity by state (IBS) estimates of each pair of samples were calculated using the Plink Distance tool (Purcell et al., 2007) and samples in pairs with IBS greater than 98% were removed as probable duplicates. After all filtering steps, around 2.9 million SNPs and 2,358 samples remained in the dataset for the ensuing genomic analyses.

Sequence data were also used to determine the sex of the chickens by heterozygosity of the sex chromosome. As females are the heterogametic (ZW) sex, samples with heterozygosity present in the sex chromosome were defined as females and all the others were assigned as homogametic (ZZ) males. The Plink tool SexCheck was used in this step (Purcell et al., 2007).

### 2.4 Phenotypic data analyses

The following fixed effect model was used in the first instance to assess the impact of multiple factors on each of the phenotypic traits described previously:

y=Xb+e (1)

where, *y* was the vector of phenotypic observations on each chicken for each trait of study; *b* was the vector of fixed effects described below; X was the incidence matrix linking *y* with the fixed effects; *e* was a vector of the random residuals (distribution N (0,I*Ve), where I was the identity matrix and Ve the residual variance of the trait).

Fixed effects tested with model [1] included the batch number, duration of the monitoring period (4 weeks in first and 8 weeks in all other batches), calendar year and month of the batch, and sex of the chicken. Following preliminary analyses, the effects of batch number and sex of the chicken were retained. The other effects assessed were confounded with the batch number, suggesting that the latter collectively accounted for all temporal, seasonal and duration effects associated with each batch. Marginal means for fixed effect levels were derived from this analysis for each trait. Additionally, the regression of each of the studied traits on body weight at the start of the monitoring period was also tested and was found significant for body weight at slaughter.

Prior to these analyses, all immune traits were log-transformed to ensure normality of distribution. Survival, a binary phenotype, was analysed by fitting a logit function to the model.

Further to the afore mentioned phenotypic traits of study, weekly body weight was analysed with mixed effect model [2] to assess chicken individuality:

y=Xb+Zu+e (2)

where, *y* was the vector of weekly body weight measured on each chicken; *b* was the vector of the fixed effects of batch number and sex of chicken; *u* was the random individual chicken effect (distribution N (0,I*Vu), where I was the identity matrix and Vu the animal variance of the trait); X and Z were the incidence matrices linking *y* to the corresponding independent variables; *e* was as in model [1].

Model [2] allowed the estimation of the proportion of total phenotypic variation of body weight attributed to differences between chickens, adjusted for the fixed effects in the model. This analysis was possible only for body weight, because it was the only trait for which repeated records were available on the same chicken.

In all cases, type I error threshold value for effect significance (*P*) was set at 0.05. The software ASReml 4.2 (Gilmour et al., 2021) was used in these analyses.

### 2.5 Genomic analyses

A principal component (PC) analysis was first conducted on the 2,358 chicken genotypes comprising 2.9 million SNPs retained for the genomic study to explore population structure. The software GEMMA 0.98.1 (Zhou and Stephens, 2012) was used and results were visualised in R (R Core Team, 2021). The genomic relationship matrix among the genotyped chickens was then generated using the same genotypic data and the software GCTA (Young et al., 2011).

Genome-wide association studies (GWAS) were subsequently performed for each phenotypic trait using mixed effect model [3].

y=Xb+Tr+Sp+Ws+Zu+e (3)

where, *y* was the vector of phenotypic observations on each chicken for each phenotypic trait of study; *b* was the vector of the fixed effects of batch number and sex of chicken; *r* was the regression on body weight at the start of the monitoring period (applied to the analysis of body weight at slaughter); *p* was the regression on the first PC; *s* was the SNP regression effect; *u* was the random polygenic effect (distribution N (0,G*Vu) where G was the genomic relationship matrix between individual chickens and Vu the polygenic variance of the trait); X, T, S, W and Z were the incidence matrices linking *y* to the corresponding independent variables; *e* was as in model [1].

The software GEMMA 0.98.1 (Zhou and Stephens, 2012) was used in this step. A Bonferroni correction was applied for multiple SNP testing to determine the genome-wide (*p* < 0.05) and suggestive (one false positive per genome scan) significance thresholds.

The genes harbouring significant SNPs identified in GWAS were annotated using the Biomart tool in Ensembl (Martin et al., 2023). The biological function of these genes was further interrogated to identify potential links with the traits under investigation using the UniProt (The UniProt Consortium, 2023), GeneCards (Stelzer et al., 2016) and OMIM (Amberger et al., 2011) databases. The same process was also applied for the closest annotated genes positioned within 100 kb downstream and upstream of the significant SNPs.

Finally, marker-based genomic and total phenotypic variance estimates were derived for each trait using mixed effect model [4]:

y=Xb+Tr+Sp+Zu+e (4)

where, *y* was the vector of phenotypic observations on each chicken for each trait of study; *b, r* and *p* were as in model [3]; *u* was the random marker-based genetic effect (distribution N (0,G*Vu), where G was the genomic relationship matrix between individual chickens and Vu the genomic variance of the trait); X, T, S and Z were as in model [3]; *e* was as in model [1].

The significance of the genomic variance estimated with model [4] was assessed with the use of the likelihood ratio test (Crainiceanu and Ruppert, 2004). The ASReml 4.2 software (Gilmour et al., 2021) was deployed for this analysis.

### 2.6 Correlations between the studied traits

Phenotypic and genomic covariances between the chicken traits were estimated in a series of bivariate statistical analyses using model [4] and the ASReml 4.2 software (Gilmour et al., 2021). Phenotypic and genomic correlations were then derived based on the corresponding trait covariance and variance estimates.

## 3 Results

### 3.1 Phenotypic analyses

**FIGURE 1:**
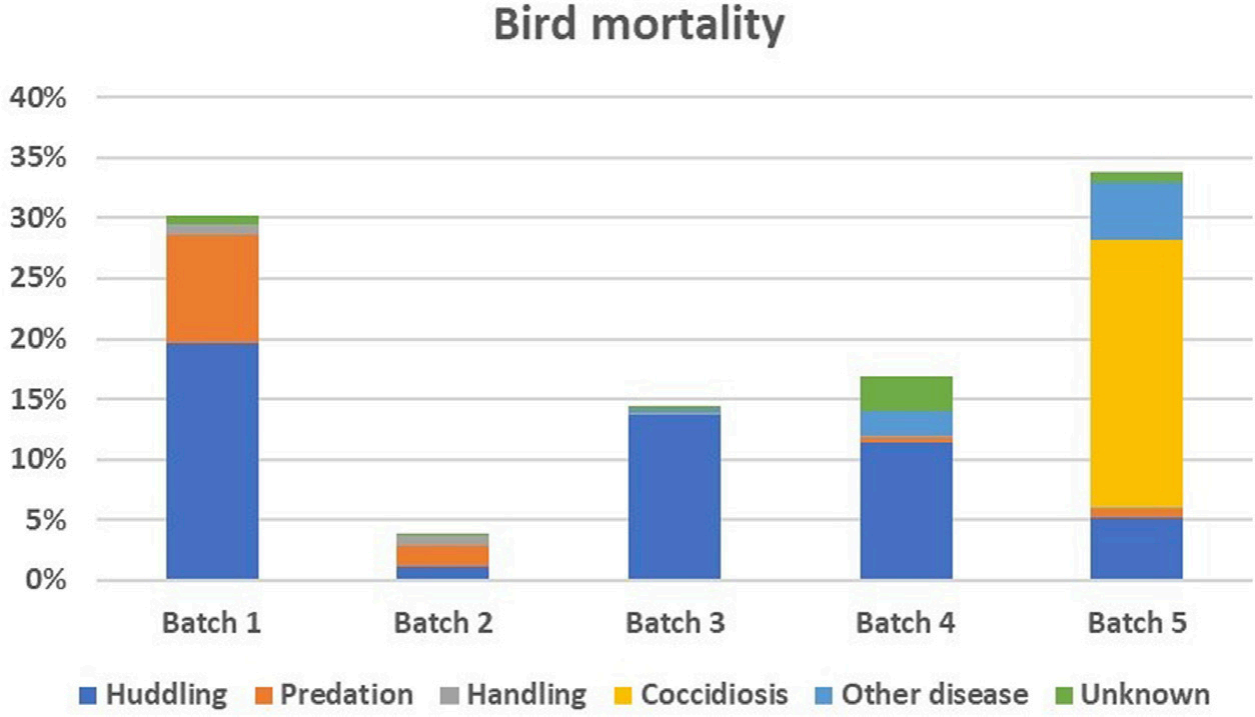
Percentage (vertical axis) of chickens that died during the outdoor monitoring period in each batch and cause of death in different colours.

Table 1 summarises the data used in the present study by batch during the outdoor monitoring period on the field. Survival and mortality rates per batch are also shown in Table 1. Figure 1 illustrates the causes of chicken death per batch. Regarding sex of the chicken, 62% of the chickens in the study were females.

**TABLE 1:** Data summary by batch.

Batch number	1	2	3	4	5	Total
Start and end dates of monitoring period^a^	21/10/2019–18/11/2019	18/12/2019–12/02/2020	13/07/2020–07/09/2020	14/10/2020–09/12/2020	24/12/2020–18/02/2021	
Average daily humidity (%)	56.5	61.4	83.6	56.7	54.5	
Average daily precipitation (mm)	0.1	0.0	10.1	0.3	0.5	
No. chickens at start of monitoring period	511	520	520	507	515	2,573
Average body weight at start of monitoring period (g)	522	541	582	581	605	566
No. chickens at end of monitoring period	359	497	445	422	339	2,062
Proportion of chickens survived	0.70	0.96	0.86	0.83	0.66	0.80
No. mortalities	152	23	75	85	176	511

^a^ Outdoor phase when chickens were reared in smallholder farm conditions.

Descriptive statistics for the studied phenotypic traits are shown in Table 2. All traits exhibited considerable variation manifested in moderate to high values of the respective coefficients of variation. The latter were highest for immune traits followed by survival, growth, and meat production.

**TABLE 2:** Descriptive statistics of the studied phenotypic traits.

Phenotypic trait	Mean	Standard deviation	Coefficient of variation (%)
Growth rate (g/d)^a^	16.24	4.99	31
Early growth rate (g/d)^a^	14.89	5.81	39
Late growth rate (g/d)^a^	15.38	5.64	37
Body weight at slaughter (g)	1,412.41	320.20	23
Breast muscle weight (g)	232.33	63.38	27
Breast muscle percentage (%)^b^	16.32%	2.05%	13
Survival rate (proportion)^c^	0.80	0.40	50
NDV titre start (normalised OD450)^d^	0.34	0.35	103
NDV titre end (normalised OD450)^d^	0.28	0.36	129
NDV titre difference (normalised OD450)^d^	−0.07	0.48	686
IgA levels start (normalised OD450)^d^	0.52	0.64	123
IgA levels end (normalised OD450)^d^	0.91	0.81	89
IgA level difference (normalised OD450)^d^	0.40	1.03	258

^a^ Growth rate during the entire outdoor monitoring period; early: weeks 1–4; late: weeks 5–8.

^b^ Percentage of body weight at slaughter corresponding to breast muscle.

^c^ Proportion of birds that survived during the monitoring period.

^d^ Immune measurements on the first and last day of the monitoring period and difference between the two, respectively.

The significance of batch number and sex of the chicken effects on each phenotypic trait is demonstrated in Table 3. Estimated marginal means of the levels of these effects are illustrated in Supplementary Figures S1 and S2 for batch number and sex, respectively.

**TABLE 3:** *p*-values demonstrating the significance of the impact of batch number and sex of chicken on the studied phenotypic traits.

Phenotypic trait	Batch	Sex
Growth rate^a^	<0.001	0.002
Early growth rate^a^	<0.001	0.010
Late growth rate^a^	<0.001	0.010
Body weight at slaughter	<0.001	<0.001
Breast muscle weight	<0.001	0.067
Breast muscle percentage^b^	<0.001	0.002
Survival rate^c^	<0.001	0.372
NDV titre start^d^	<0.001	0.040
NDV titre end^d^	<0.001	0.356
NDV titre difference^d^	<0.001	0.518
IgA levels start^d^	<0.001	0.271
IgA levels end^d^	<0.001	0.005
IgA level difference^d^	<0.001	0.119

^a^ Growth rate during the entire outdoor monitoring period; early: weeks 1–4; late: weeks 5–8.

^b^ Percentage of body weight at slaughter corresponding to breast muscle.

^c^ Proportion of birds that survived during the monitoring period.

^d^ Immune measurements on the first and last day of the monitoring period and difference between the two, respectively.

Batch number had a strongly significant effect (*p* < 0.05) on all phenotypic traits. This effect encompasses all temporal, seasonal, spatial, cohort and other external factors associated with conditions that applied to each group of chickens reared together. The significance of the batch number effect warranted its inclusion in the subsequent genomic analyses to properly account for this important source of variation.

Sex of the chicken significantly (*p* < 0.05) affected growth and meat production traits (Table 3). Male chickens grew faster, were heavier at slaughter and had greater breast muscle weight, although the latter was marginally not significantly different from the females’ (*p* = 0.07). On the other hand, breast muscle weight as a proportion of body weight at slaughter was greater in female than male chickens (*p* < 0.05). Female chickens were also associated with significantly (*p* < 0.05) higher IgA levels at the end of the outdoor monitoring period. On the contrary, the NDV titre at the beginning of the monitoring period was significantly (*p* < 0.05) greater in males. Sex did not affect the other immune traits of the study. Sex of the chicken did not affect survival during the monitoring period, either.

In addition to the phenotypic traits in Table 3, chicken body weight measured weekly was analysed separately to determine the effect of bird individuality. This was possible only for this trait where repeated records were available on each chicken. After adjusting for the batch number and sex of the chicken effects, 65.3% (*p* < 0.05) of the total phenotypic variance in body weight throughout the outdoor growth phase was attributed to the individuality of the bird. In the ensuing genomic analysis of the present study, progression of weekly body weights was captured in the growth rate phenotypes.

### 3.2 Genotyping by sequencing and imputation accuracy

Sequencing and imputation quality was confirmed using the two approaches previously described. The sequential down-sampling and imputation approach using genomes from the imputation panel revealed an average concordance between imputed and actual genotypes of 99% across all sites. Comparison between the imputed low-pass and the 20X coverage sequences of the 20 samples from the present study showed a concordance of 93% and 97% across all sites and the sites with a genomic score greater than 0.90, respectively. The latter genotypes were retained for the genomic analysis. Compared to the first, the second approach led to somewhat lower correlations probably due to additional sources of accumulating errors in the two separate sequencing runs. Nevertheless, the two approaches combined confirm that the sequencing and imputation process was of high accuracy and that the imputed genotypes were reliable.

### 3.3 Genomic analyses

The eigenvalues of the first 20 PCs are illustrated in Supplementary Figure S3. The eigenvalue of the first PC was more than twice the size of the each of the others, reflecting the same relationship in the variance magnitude explained by each PC. Plotting the first two PCs revealed two clusters (Supplementary Figure S4) that did not directly correlate with batch number or sex of the chickens and may have been due to the chicks emanating from multiple groups of parental lines. To account for this genotypic population structure, the first PC was included in the model of the ensuing genomic analyses (Hoffman, 2013). In addition, inclusion in the model of the genomic relationship matrix between individual chickens accounted for any kinship structure in the studied population (Hoffman, 2013).

Genome-wide association studies (GWAS) revealed a total of 15 SNPs significantly correlated with 10 phenotypic traits associated with chicken growth, meat production, survival, and immunity (Table 4; Supplementary Figures S5 and S6). Among these SNPs, the only genome-wide significant (*p* < 0.05 post Bonferroni correction) association was of a SNP on chromosome 10 related with growth rate during the entire monitoring period. The same SNP had a suggestive significant effect (one false positive per genome scan) on early growth rate during the first 4 weeks but did not correlate with the later part of the outdoor growth phase. All other associations shown in Table 4 were significant at the suggestive level.

**TABLE 4:** Single Nucleotide Polymorphisms (SNPs) with a significant effect on the phenotypic traits identified in genome-wide association studies.

Phenotypic trait	Chromosome no.	Position (kb)	*p*-value	Gene	Distance of SNP (bp)	Annotated gene function
Growth rate^a^	3	10,567,409	2.86E-07	CEP68	84	Centromere function, protein localisation
Growth rate	10	3,415,151	1.38E-08	HMG20A	139	Neuronal differentiation, protein binding activity, regulation of gene expression
Early growth rate^a^	10	1,839,061	3.77E-08	NEO1	7,981	Multi-functional cell surface receptor regulating cell adhesion in many diverse developmental processes
Early growth rate	10	3,415,151	2.25E-07	HMG20A	139	Neuronal differentiation, protein binding activity, regulation of gene expression
Early growth rate	28	3,788,734	3.81E-07	CCDC124	intron	Enables RNA binding activity
Late growth rate^a^	1	1,308,946	2.07E-07	STRIP2	exon	Cell migration, cytoskeleton organisation, regulation of cell shape
Body weight at slaughter	2	30,658,369	7.25E-08	RAPGEF5	intron	GTPase, signal transduction, RAS activator; associated with hypoparathyroidism is humans
Breast muscle percentage^b^	5	58,058,873	1.26E-07	NIN	intron	Centrosomal function, centriole organisation; associated with dwarfish, low birth weight and growth retardation in humans
Survival rate (coccidiosis)^c^	8	7,444,392	3.54E-07	NPL	2,156	Controls the cellular concentration of sialic acid
NDV titre start^4^	1	101,630,135	1.21E-07	lncRNA	62,085	Unknown
NDV titre start	4	86,115,915	1.92E-07	HRH2-like	4,290	Mediates histamines
NDV titre difference^4^	1	15,572,290	3.87E-07	lncRNA	intron	Unknown
NDV titre difference	3	39,872,108	3.19E-07	lncRNA	intron	Unknown
IgA levels end^4^	1	130,165,242	3.86E-07	STAG3	intron	Regulates the cohesion of sister chromatids during cell division
IgA level difference^d^	9	10,058,375	1.35E-07	TFDP2	intron	Mediates both cell proliferation and apoptosis
IgA level difference	5	23,397,007	3.86E-07	PHF21A	intron	Represses transcription of neuron-specific genes in non-neuronal cells

^a^ Growth rate during the entire outdoor monitoring period; early: weeks 1–4; late: weeks 5–8.

^b^ Percentage of body weight at slaughter corresponding to breast muscle.

^c^ Proportion of birds that survived the coccidiosis outburst in the fifth batch.

^d^ Immune measurements on the first and last day of the monitoring period and difference between the two, respectively.

Markers associated with chicken growth and meat production traits were located on chromosomes 1, 2, 3, 5, 10 and 28. There were other SNPs on chromosomes 1, 3 and 5 also associated with immune traits but they were not linked to the SNPs on the same chromosomes that correlated with growth rate and meat production. Additional SNPs affecting immune traits were located on chomosomes 4 and 9 (Table 4).

Our study initially revealed no SNPs associated with overall chicken survival across all batches. We then took a closer look at the chickens that died or survived coccidiosis during the fifth batch, where coccidiosis accounted for the majority of the losses (Figure 1). A suggestive significant SNP was identified on chromosome 8 that was associated with survival to coccidiosis (Table 4).

A small proportion of trait variance was explained by each one of the significant SNP markers ranging from 1.4% to 1.7%. In certain cases, multiple unlinked SNPs were identified that were correlated with the same trait. For example, the three SNPs affecting early growth rate collectively accounted for 4.3% of the trait variance. Similarly, two SNPs affected overall growth rate explaining 3.1% of the variance, whereas 2.3%–3.0% of the trait variance was attributed to multiple SNPs associated with chicken immune profile traits.

Further interrogation of the regions around the significant SNP markers revealed a number of annotated genes summarised in Table 4.

Marker-based genomic variance estimates were derived for the studied phenotypic traits. This source of variation emanates collectively from all SNPs (ca. 2.9 million) retained in the genomic analysis. Genomic and phenotypic variance estimates and the ratios of the two are shown in Table 5. Marker-based genomic variance estimates were significantly greater than zero (*p* < 0.05) for overall growth rate, early growth rate in the first 4 weeks of the monitoring period, body weight at slaughter, breast muscle weight, breast muscle percentage and IgA levels on the last day of the monitoring period. The corresponding ratio estimates for these traits ranged from 0.082 to 0.131. Furthermore, a statistical trend (0.05 < *p* < 0.10) pertained to the genomic variance of NDV titre and IgA level difference between the first and last day of the monitoring period; respective ratio estimates were 0.047 and 0.061 (Table 5).

**TABLE 5:** Marker-based genomic and phenotypic variance estimates and ratio of the two for the studied phenotypic traits; standard errors in parentheses.

Phenotypic trait	Genomic variance^a^	Phenotypic variance	Ratio
Growth rate (g/d)^b^	2.40 (0.96)*	18.33 (0.51)	0.131 (0.059)
Early growth rate (g/d)^b^	2.44 (1.23)*	21.73 (0.71)	0.112 (0.056)
Late growth rate (g/d)^b^	1.78 (2.00)	24.39 (1.12)	0.073 (0.082)
Body weight at slaughter (g)	7,143 (2,748)*	61,382 (2,040)	0.116 (0.048)
Breast muscle weight (g)	295 (132)*	2,815 (95)	0.105 (0.050)
Breast muscle percentage (%)^c^	0.003 (0.002)*	0.025 (0.001)	0.120 (0.061)
Survival rate (proportion)^d^	0.036 (0.889)	3.336 (0.937)	0.011 (0.099)
NDV titre start (normalised OD450)^e^	0.002 (0.004)	0.096 (0.003)	0.022 (0.038)
NDV titre end (normalised OD450)^e^	0.002 (0.001)	0.040 (0.001)	0.041 (0.034)
NDV titre difference (normalised OD450)^e^	0.006 (0.004)‡	0.124 (0.003)	0.047 (0.033)
IgA levels start (normalised OD450)^e^	0.005 (0.018)	0.326 (0.011)	0.016 (0.052)
IgA levels end (normalised OD450)^e^	0.043 (0.025)*	0.524 (0.017)	0.082 (0.048)
IgA level difference (normalised OD450)^e^	0.050 (0.034)‡	0.814 (0.020)	0.061 (0.041)

^a^ Significance of genomic variance estimate based on the likelihood ratio test: *p < 0.05; ‡ 0.05 < *p* < 0.10.

^b^ Growth rate during the entire outdoor monitoring period; early: weeks 1–4; late: weeks 5–8.

^c^ Percentage of body weight at slaughter corresponding to breast muscle.

^d^ Proportion of birds that survived during the monitoring period.

^e^ Immune measurements on the first and last day of the monitoring period and difference between the two, respectively.

### 3.4 Correlations between the phenotypic traits

Phenotypic correlations between the studied traits are included in Supplementary Table S2. Expectedly, moderate to strong positive correlations were found between growth and meat production traits indicative of faster growing birds having greater body weight at slaughter and greater breast muscle weight. These correlation estimates ranged from 0.447 to 0.921 and were significantly different from zero (*p* < 0.05).

Phenotypic correlations of growth and meat production traits with survival and immune phenotypes were low (Supplementary Table S2). The highest such estimate was a positive phenotypic correlation of 0.150 between early growth rate (first 4 weeks of the monitoring period) and survival, suggesting that faster growing chickens at the onset of their exposure to the outdoor environment might have coped better with the external challenges. Although this estimate was significantly different from zero (*p* < 0.05), biologically suggests presence of a very small proportion of common variance between the traits.

Phenotypic correlations between survival and the immune measurements at the start of the monitoring period were low. It was not possible to estimate the correlation with immune traits at the end of the monitoring period since the latter phenotype was only available for birds that survived (Supplementary Table S2). Phenotypic correlations between the NDV specific serum titres and mucosal IgA phenotypes were practically zero, indicating that the two traits capture distinct parts of the chicken immune profile.

Decomposing the phenotypic covariance into statistically significant (*p* < 0.05) genomic and residual components was possible only in a few cases. Corresponding estimates of genomic correlations are summarised in Table 6 and pertain to growth and meat production traits.

**TABLE 6:** Statistically significant (*p* < 0.05) genomic correlations between phenotypic traits.

Trait 1	Trait 2	Estimate	Standard error
Growth rate^a^	Early growth rate^a^	0.91	0.12
Growth rate	Body weight at slaughter	0.96	0.08
Growth rate	Breast muscle weight	0.88	0.14
Early growth rate	Breast muscle weight	0.89	0.23
Body weight at slaughter	Breast muscle weight	0.84	0.11
Breast muscle weight	Breast muscle percentage^b^	0.81	0.29

^a^ Growth rate during the entire outdoor monitoring period; early: weeks 1−4.

^b^ Percentage of body weight at slaughter corresponding to breast muscle.

## 4 Discussion

The present study aimed to assess multiple phenotypic traits associated with growth, meat production, systemic and mucosal immunity, and survival of tropically adapted commercial chickens raised in smallholder farm conditions in Ethiopia. The study also examined the genetic background of these phenotypic traits and identified genomic markers and genes that may correlate with the phenotypes and inform selection practices in future breeding programmes. To achieve our objectives, we implemented a large-scale study design comprising more than 2,500 chickens and 13 phenotypic traits measured on each bird. This was made possible by emulating study conditions at the ILRI poultry facility in Addis Ababa, Ethiopia, and individually monitoring chicken performance during their outdoor growth phase.

To our knowledge, the present is the largest reported phenotypic and genomic study of poultry raised in smallholder farm conditions. It is also the first such study of Sasso chickens raised outdoors that reports genomic markers and genes associated with a range of phenotypic traits of potential interest to smallholder farmers.

### 4.1 Phenotypic characterisation of chicken performance

We observed extensive phenotypic variation in all chicken traits of the study. We anticipate a proportion of this to reflect genetic variability that can be used to identify selection sites in breeding programmes. Indeed, when repeated body weight records on the same chicken were analysed, approximately two-thirds of the observed phenotypic variation was due to differences among individual birds and one-third was attributed to external factors. The former reflects trait repeatability and may be considered in developing tools for better on-farm chicken management and selection practices.

Across the five batches, 20% of the chickens died during the outdoor monitoring period (Table 1). Most deaths (59%) occurred during the first week of the monitoring period, probably associated with the stress from transitioning from the indoor brooding environment to the challenges of the outdoor conditions. Lower mortality rates afterwards suggested that chickens were gradually adapting to the change and the new environment. This transition reflects the current practice in the field, where breeding companies sell chickens at the age of 56 days to smallholder village farms.

There were multiple causes of chicken mortality illustrated in Figure 1. In the first batch, increased mortality rates were attributed to predators visiting the paddocks with birds panicking in the outdoor sheds and huddling together and suffocating each other (Figure 1). To varying levels, predation and huddling remained an issue in the subsequent batches. Elevated mortality rates were also observed in the fifth batch, mainly due to a coccidiosis outbreak during the monitoring period of the outdoor phase that accounted for nearly two-thirds of the deaths. Variation in predation and disease has been identified as a key constraint to chicken production in smallholder farm conditions (Aman et al., 2017). This may be due to both random environmental challenges in the field and variability in chicken capacity to elude predators and pathogens. In the present study, this unpredictability in occurrence was captured by the batch number effect that was included in all models of analysis.

In addition to accounting for statistical noise due to random circumstances associated with each batch, the batch number effect also combined the impact of systematic variation of batch duration and the temporal effect of calendar year and season. It was not possible to further disentangle the effect of each of the other factors due to confounding with the batch number. For example, all batches except for the third took place during the dry season characterised by mild to high temperatures and moderate humidity (NMA, 2023). The original design was to have all batches in the dry season to ensure experimental homogeneity regarding seasonal variation. However, we had to adapt to COVID-19 pandemic restrictions and bird procurement issues and include a batch in the wet and rainy season (third batch, July-September), when average daily precipitation was 10.1 mm compared to less than 0.5 mm in the other batches, while average air humidity was 84% and 55%–61%, respectively (Table 1; NMA, 2023). These differences might explain the decreased growth rate in the third batch, especially during the first weeks of the outdoor monitoring period (Supplementary Figure S1) when the birds were still trying to adapt to the external environmental challenge. Therefore, the statistical significance of the batch number effect on all studied phenotypic traits (Table 3) came as no surprise. Fitting the batch number effect in the model allowed us to effectively pool data from all batches together in a joint analysis.

Sex of the chicken affected the growth and meat production traits (Table 3). Male chickens grew faster and had greater body weight at slaughter by 8%–10% and larger and heavier breast muscle by approximately 5% compared to females. This is broadly in agreement with earlier reports on indigenous and tropically adapted chicken ecotypes (Osei-Amponsah et al., 2012; Jahan et al., 2015; Tola et al., 2022). Sex differences in the body weight between female and male Sasso chickens were also observed in on-station studies of intensively raised birds (Yakubu and Ari, 2018; Bamidele et al., 2020). The proportion of body weight at slaughter corresponding to breast muscle in the present study was higher in females than males by 2%, suggesting potentially more efficient energy partitioning to meat and muscle development during the outdoor growth phase. The latter merits further research. Previous studies reported minor differences between sexes in breast meat percentage in topically adapted chickens in Bangladesh (Jahan et al., 2015) and higher male performance in indigenous Nigerian chickens (Isidahomen et al., 2012). Such results may be connected to sexual dimorphism resulting from hormonal differentiation between the two genders (Semakula et al., 2011).

Sex of the chicken was also associated with two of the six immune traits included in our study. Female chickens had higher IgA levels at the end of the outdoor monitoring period, although they did not differ from males at the beginning of monitoring. This result potentially suggests that females may have a greater capacity for overall mucosal response to infection when exposed naturally to free-ranging conditions. On the other hand, slightly but significantly higher NDV titres were observed in males at the beginning of the monitoring period. Since birds had not yet been exposed to the outdoor environment at that stage, this result suggests a likely higher response to vaccination during the indoor brooding phase in male than female chicks. This is opposite to earlier reports on Kenyan village chickens (Njagi et al., 2010) and tropically adapted and local ecotypes in Ghana (Osei-Amponsah et al., 2013) at the same stage of development. No differences between sexes were observed in NDV titre records at the end of the monitoring period and variation between birds was the same in both sexes, indicating a possibly similar response to the naturally occurring pathogen. This agrees with similar studies on indigenous chickens in Ghana (Tudeka et al., 2022) and Nigeria (Adenaike et al., 2020), but is contrary to reports by Njagi et al. (2010) on village chickens in Kenya. Differences between studies may be attributed to a multitude of factors not least pertaining to data, design, definition and methods of deriving the immune phenotypes, and statistical analysis. Sex differentiation of immune response to other pathogens in village chickens has been previously reported (Bettridge et al., 2014; Psifidi et al., 2016). There is evidence that the sex chromosomes may be implicated in immune response of chickens (Boa-Amponsem et al., 1997; Garcia-Morales et al., 2015; Psifidi et al., 2016) leading to an expectation of certain sex differentiated immune phenotypes. In any case, sex profiles of immune response are likely to differ between distinct chicken breeds and ecotypes, so the results presented here pertain primarily to the studied population.

In our study, phenotypic characterisation of chicken performance focused on outdoors growth, meat production, immune response, and survival. Such phenotypes would be of interest to smallholder farmers and important to improve with appropriate management and breeding strategies. Moreover, village chickens raised outdoors are exposed to environmental challenge including weather variability and climate change (Tiruneh and Tegene, 2018). Extreme weather events and increased weather volatility will impact on chicken performance directly and indirectly by altering food and water resources of scavenging chickens (Alemayehu and Woldeamlak, 2017; Abioja and Abiona, 2021). Future studies should focus on the targeted and detailed phenotypic characterisation of individual chicken response to environmental stressors, including climate change, looking at novel phenotypes reflecting the resilience of chicken performance under smallholder farm conditions. Future studies should also aim to dissect bird genotype by environment interactions and their impact on assessing chicken performance in the tropics.

### 4.2 Genomic characterisation of chicken performance

Collectively, our results suggest presence of genetic variation in phenotypes associated with the studied trait categories, namely, growth rate, meat production, immune profile, and survival. This is supported by a combination of GWAS results (Table 4) and marker-based genomic variance estimates (Table 5), where there is at least one significant outcome for each trait. Results of GWAS detected single-SNP signals associated with the phenotypes, whereas marker-based genomic variance estimates portrayed the overall polygenic effect captured by the 2.9 million SNPs spread across the whole chicken genome.

We report here individual SNPs on chromosomes 1, 2, 3, 5, 10 and 28 linked to growth and meat production traits (Table 4). We also report distinct markers on chromosomes 1, 3, 4, 5, eight and nine related with the studied immune traits and chicken survival (Table 4). All these markers were located either within or directly upstream of these genes. An earlier GWAS on NDV titres at the end of the monitoring period conducted on chickens of the fourth batch of the present study, reported two SNPs with suggestive significant associations (Girma et al., 2023) These SNPs, however, did not maintain their suggestive significance here, probably because of data size difference and the present being an across-batch analysis accounting for the batch number effect.

Interrogation of the regions harbouring the significant SNPs of the present study revealed that the markers were located either within or directly upstream of annotated genes (Table 4). A significant SNP associated with breast muscle percentage was located within the *NIN* gene, which has been reportedly implicated in developmental delay (Dauber et al., 2012) and short stature and skeletal deformities (Grosch et al., 2013) in humans, as well as embryonic development in goats (Berihulay et al., 2022; Getaneh and Alemayehu, 2022). This suggests a potential role of the *NIN* gene in growth traits of other species, too. Furthermore, the gene *HRH2-*like contained a SNP correlated with NDV titres. Studies on knockout mice models have shown a complex effect of this gene on the immune system, including *HRH2* knockout mice having lower levels of antigen-specific Immunoglobulins G and E (Jutel et al., 2001). The gene has been linked to histamine receptor signalling and cytokine production reflecting antigen-presenting cell activity in mice (Teuscher et al., 2004), but its potential role in chicken response to vaccination and infectious disease challenge remains unknown and merits further investigation. Moreover, some of the markers associated with NDV titres were located within or nearby long noncoding RNA (lncRNA) in the present study. Previous research has demonstrated the diverse function of lncRNAs and implicated them as potential biomarkers of human immune response and health (Li et al., 2021; Abdolmajid et al., 2023), although the exact role of most lncRNAs is still unknown (Ransohoff et al., 2018).

We acknowledge the challenge of conducting GWAS on crossbred data, where linkage disequilibrium is small, thereby hindering the detection of individual SNPs. At the same time, we deployed whole-genome sequencing and implemented a strict Bonferroni correction for repeat testing, which may have actually set the significance threshold too low. Under these conditions, SNPs that attain post-Bonferroni statistical significance may be viewed as rather promising, especially when they are located within or very close to annotated genes, as was the case here. Previous GWAS studies on commercial crosses have attested to the challenge and utility of such analyses and results (Gao et al., 2021; Silva et al., 2019; Zhang et al., 2016).

The proportion of trait variance explained by each significant marker was notably low (<2%), suggesting a likely polygenic genetic control of the studied phenotypic traits, where multiple genes are involved, each with a low or modest effect. Such traits are amenable to genomic selection based on estimates of the overall genomic merit of selection candidates, which is widely used in the global poultry industry (Wolc et al., 2016). Information on individual SNPs and candidate genes presented here may be used to increase the accuracy of genomic predictions and selection for the improvement of traits under largely polygenic control (Iheshiulor et al., 2017). Future research should focus on tracing the presence of these SNPs and genes to the parental lines of the Sasso T451A cross and evaluating them (Ibánẽz-Escriche et al., 2009) towards informing and enabling accurate genetic selection at the parental level for improved performance of future progeny in smallholder farms.

The genomic markers and associated genes reported in the present study are quite unique and do not coincide with SNPs and genes reported in previous studies on other tropically adapted and indigenous African chicken ecotypes for productivity (Psifidi et al., 2016), immune performance (Psifidi et al., 2016; Banos et al., 2020), and health (Fleming et al., 2016), suggesting the existence of distinct genetic profiles in different chicken populations. Thus, our results pertain and may contribute to the genetic improvement of the specific tropically adapted chicken type in the studied rearing conditions.

Genomic correlations between growth rate, body weight, and breast muscle weight were close to unity (Table 6), suggesting that these traits are under similar genetic control. Although different SNP markers were associated with each of these traits (Table 4) the overall genomic correlation estimates based on 2.9 million SNPs captured a major part of the polygenic covariance between the traits. The significance of this outcome is that genetic improvement on any of these traits will also lead to improvement of the others. Interestingly, the genomic correlation between early and late growth rates was not different from zero, suggesting that different genes may affect the two traits. Late growth rate was not correlated genomically with meat production traits, either. These results imply that the genetic profiles of growth and meat production in the studied population are probably linked to the early adaptation of chickens at the time they transition from indoor brooding to outdoor rearing conditions.

Collectively, our results showed no genetic antagonism of chicken production manifested in growth rate, body weight and breast muscle weight, with fitness traits exemplified by immune measurements and bird survival. Different genetic markers were associated with the two sets of traits (Table 4). Genomic correlations were not different from zero meaning that phenotypic correlations (Supplementary Table S2) between the two groups of traits are mainly due to common environmental effects. Importantly, these results suggest that genetic improvement for enhanced production would not be expected to compromise fitness of the chickens. Interestingly, lack of genetic antagonism between production and immune response and health was also reported in previous studies on Ethiopian Horro and Jarso indigenous chickens raised in village conditions (Psifidi et al., 2016; Banos et al., 2020).

In conclusion, our results provide new insights into the performance of tropically adapted commercial chickens reared outdoors in free-ranging village conditions. Results may contribute to the optimisation of breeding programmes catering to the needs of smallholder farmers. We envisage these results to inform future selection practices of breeding parents for enhanced performance of their progeny when the latter are reared in smallholder village farms in sub-Saharan Africa.

## Data Availability

The data presented in the study are deposited in the following repository: University of Edinburgh. Centre For Tropical Livestock Genetics and Health https://doi.org/10.7488/ds/7718.
